# Reconstitution of Membrane-tethered Minimal Actin Cortices on Supported Lipid Bilayers

**DOI:** 10.3791/63968

**Published:** 2022-07-12

**Authors:** Darius Vasco Köster, Abrar Bhat, Sankarshan Talluri, Satyajit Mayor

**Affiliations:** 1 Centre for Mechanochemical Cell Biology and Division of Biomedical Sciences, Warwick Medical School, University of Warwick; 2 National Centre for Biological Sciences, Tata Institute of Fundamental Research

## Abstract

The surface of a living cell provides a versatile active platform for numerous cellular processes, which arise from the interplay of the plasma membrane with the underlying actin cortex. In the past decades, reconstituted, minimal systems based on supported lipid bilayers in combination with actin filament networks have proven to be very instrumental in unraveling basic mechanisms and consequences of membrane-tethered actin networks, as well as in studying the functions of individual membrane-associated proteins. Here, we describe how to reconstitute such active composite systems *in vitro* that consist of fluid supported lipid bilayers coupled via membrane-associated actin-binding proteins to dynamic actin filaments and myosin motors that can be readily observed via total internal reflection fluorescence microscopy. An open-chamber design allows one to assemble the system in a step-by-step manner and to systematically control many parameters such as linker protein concentration, actin concentration, actin filament length, actin/myosin ratio, as well as ATP levels. Finally, we discuss how to control the quality of the system, how to detect and troubleshoot commonly occurring problems, and some limitations of this system in comparison with the living cell surface.

## Introduction

The plasma membrane of a living animal cell constantly interacts with the adjacent actin cytoskeleton, and together they form an active composite material that fulfills a multitude of cellular functions^1, 2^. To study processes at this lipid membrane-actin interface, the reconstitution of cytoskeletal networks on top of supported lipid bilayers (SLBs) has proven to be very helpful. This minimal system approach allows the precise control of cytoskeleton network components and lipid composition. Compared to the free-standing lipid membranes of giant unilamellar vesicles, the planar geometry of SLBs allows efficient use of state-of-the-art microscopy techniques such as super-resolution^3,4^, total internal reflection fluorescence (TIRF)^5, 6, 7^, or interferometric scattering^8^to study the spatial organization and dynamics of cytoskeletal networks. TIRF provides the highest contrast for fluorescently labeled components, since the signal of unbound labeled molecules in the solution contributing to the background signal is minimal.

**Figure 1:**
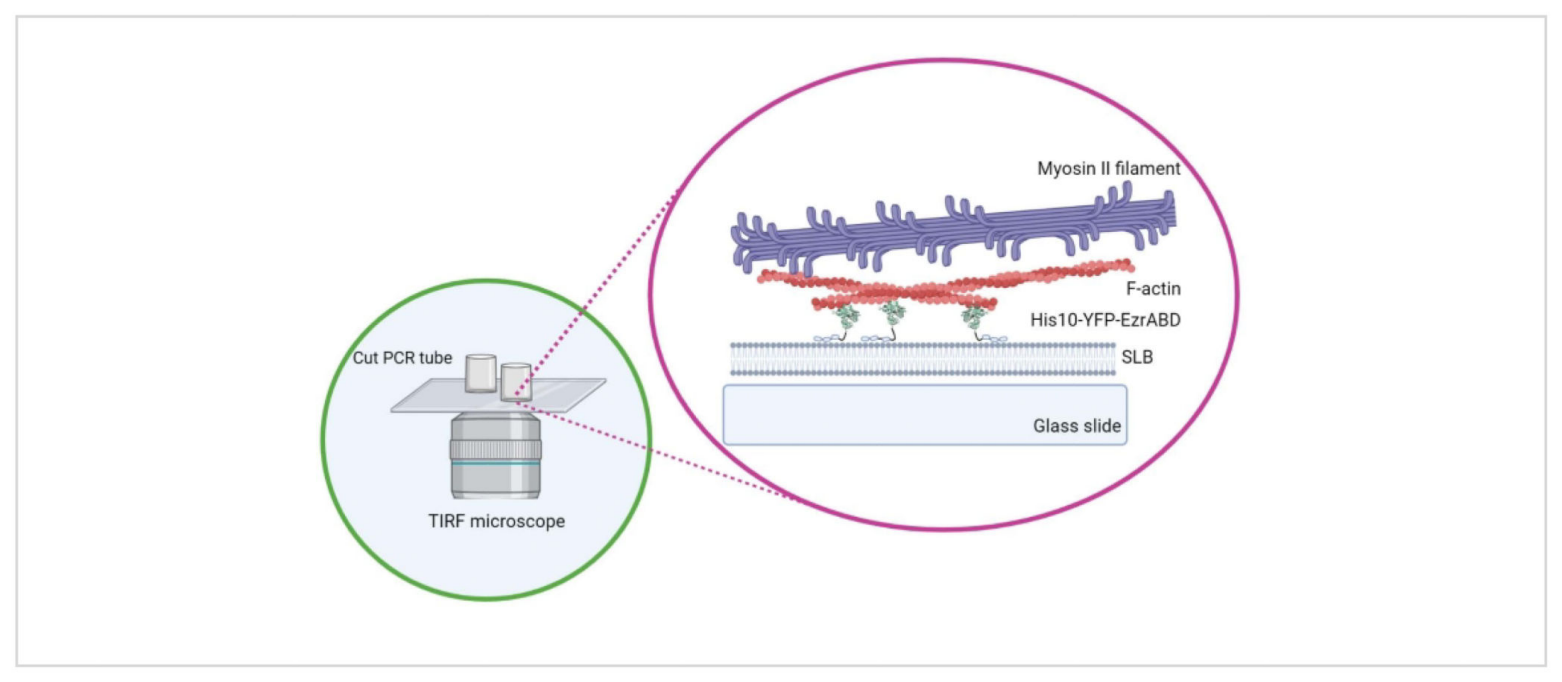
Schematic of the *in vitro* actin-membrane active composite system. Created with Biorender. Please click here to view a larger version of this figure.

Here, we describe a basic protocol for the formation of actomyosin networks tethered to supported lipid bilayers, which are widely used in the field to study the physics of active, quasi-2D networks^9, 10, 11^and their effect on membrane organisation^3, 5, 12, 13, 14, 15, 16^(Figure 1). This approach is not limited to actin-based networks but can also be adapted easily to explore microtubules, intermediate filaments, or composite networks of mixed nature and to study a variety of interactions between lipid membrane proteins and cytoskeletal components using surface-sensitive microscopy methods.

To keep this protocol focused, we have excluded a detailed description of the purification and labeling of actin and myosin proteins or details about how to tune and control the contractility and organization of actomyosin networks. One should refer to other protocols that are published alongside this one in the JoVE Methods Collection, In Vitro Reconstitution of Cytoskeleton Networks for Biomaterials, Biophysics and Active Matter Research^17^.

## Protocol

### 1 Reagents and equipment

**Figure 2:**
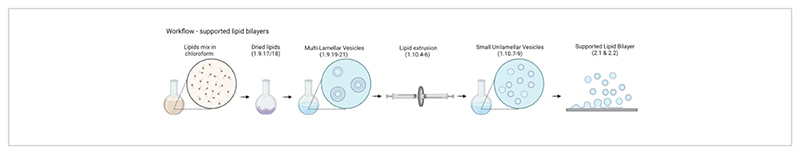
Schematic showing the workflow from preparing multilamellar vesicles and small unilamellar vesicles to the formation of supported lipid bilayers. Created with Biorender. Please click here to view a larger version of this figure.

1. Prepare fresh buffers as listed in Table 1. Use ultrapure, deionized water with a resistivity of 18.2 MΩ·cm at 25 °C. Sterilize all the buffers by passing them through 0.22 μm filters under a vacuum. Degas the buffers used for column chromatography.
2. Purify skeletal muscle actin as described earlier^18, 19^. Add 20% glycerol to the final purified G-actin solution and make aliquots of 500 μL (for labeling or bulk experiments) and 10 μL (for individual experiments) volume. Flash freeze the aliquots by dipping the tubes in liquid nitrogen for 30 s and then store them at −80 °C for up to 18 months.
  **NOTE:** Alternatively, purified actin or acetone powder can be purchased commercially.
3. Label purified skeletal muscle G-actin with any fluorescent maleimide dyes as described earlier^5^. Determine the concentration and the degree of labeling of the protein by spectrophotometry using corrected A_290nm_for actin (εactin = 26,600 M^-1^cm^-1^) and A_λmax_of the dye. Make aliquots of 10 μL and flash freeze by dipping the tubes in liquid nitrogen for 30 s and store at −80 °C for up to 18 months.
  **NOTE:** Labelling with lysine-conjugating NHS-esters will create non-functional actin and should be avoided.
4. Purify skeletal muscle myosin II by following the protocol^20^. Run SDS-PAGE using 10% polyacrylamide gel followed by Coomassie staining to determine the purity level of the protein^21^. Store purified skeletal muscle myosin-II at −20 °C in liquid form in myosin II buffer with 50% glycerol.
  **NOTE:** The stored myosin II can be used for up to 2 years.
5. Label purified myosin-II with any fluorescent maleimide dyes as described earlier^5^. Avoid labeling myosin motors with NHS-esters dyes. Determine the concentration and degree of labeling by spectrophotometry using corrected A_280nm_of myosin II and A_λmax_of the dye. Store the recycled myosin II (dark or labeled) at 4 °C and use within 6 weeks.
6. Purification of capping protein
  1. Obtain murine capping protein by following an earlier protocol^22^. Run SDS-PAGE using 10% polyacrylamide gel followed by Coomassie staining to determine the purity level of the protein. Measure the concentration using A_280nm_of capping protein (ε_CP_= 99,530 M^-1^cm^-1^).
  2. Add 20% glycerol to the protein solution and make 5 μL aliquots in 200 μL PCR tubes. Plunge the tubes into liquid nitrogen and store them at −80 °C for up to 2 years.
    **NOTE:** Capping protein activity is checked by polymerizing fixed amounts of fluorescent G-actin in the presence of different capping protein amounts. The filaments are then imaged under a microscope, and their length distribution is quantified. The higher the relative concentration of capping protein, the shorter the actin filament distributions. See Köster et al.^5^.
7. Express a fluorescent membrane-actin linker protein, e.g., for this protocol use 10xHis-YFP-EzrinABD (HYE), express it in Bl21DE3* *Escherichia Coli*, and purify as described earlier^23^. Determine the concentration of the protein by spectrophotometry.
8. Store the protein in small aliquots in gel-filtration chromatography buffer (or any other appropriate buffer) with 20% glycerol at −80 °C. Under these conditions, the protein is stable for over 2 years.
  **NOTE:** The choice of actin-membrane linker protein and fluorescent marker depends on the type of question one is addressing. A wide range of lipid-linking strategies have been developed in the past years including Histidine-tagged proteins^24^, biotin-streptavidin^25^, and single-stranded DNA^26^.
9. Preparation of multi-lamellar vesicles (MLVs)
  **NOTE:** The workflow from MLVs to supported lipid bilayers is depicted in Figure 2.
  1. Place 5-10 amber glass vials in a 200 mL glass beaker. Fill the beaker with 2% cleaning solution, just enough to submerge the glass vials. Sonicate them in a water bath for 30 min at full pulse and 65 °C.
  2. Take out the vials from the solution and rinse them thoroughly with distilled water. Place the vials in a glass beaker containing 2 N NaOH and sonicate for 20 min. No heating is required during this step.
  3. Take out the vials from NaOH solution and rinse thoroughly with distilled water. Dry the vials inside a hot air oven set at 65 °C for 2 h or longer.
  4. Store the cleaned vials in a clean beaker sealed with transparent film for up to 6 weeks.
    CAUTION: Perform the following steps inside a chemical fume hood. Handle chloroform and the lipid solutions with gas-tight Hamilton glass syringes to avoid contamination by plastic.
  5. Rinse the Hamilton syringes and a few amber glass vials multiple times with pure chloroform. Take the lipid powder stored in glass ampules from the −20 °C freezer and add adequate volumes of chloroform to dissolve the lipid powders to concentrations of 10-25 mg/mL.
  6. Transfer the solution from the ampule to a freshly cleaned amber glass vial and label it appropriately. Perform this step on ice to reduce evaporation of chloroform.
  7. Make a DOPC stock solution with a concentration of 10-25 mg/mL and DGS-NTA-Ni^2+^with a concentration of 1-10 mg/mL.
  8. To make a working lipid mix, take a clean glass vial and rinse it 2x with chloroform. Add 300 μL of pure chloroform to the vial to serve as base for better mixing of the components. This will not affect the final concentrations of the lipids as all the chloroform will be dried out in the next steps.
  9. Add measured volumes of stock lipid solutions to the vial to make the desired working lipid mixes. The target lipid concentration in lipid rehydration buffer is 4 mM. Dry the lipid mixture under a slow stream of N_2_gas inside the chemical hood at room temperature. This step can take up to 30 min for each vial.
  10. After all the solvent has dried out, vacuum-desiccate the lipid film for >2 h at room temperature to remove any traces of chloroform left. Resuspend the desiccated lipid mix in lipid rehydration buffer for a final lipid concentration of 4 mM.
  11. Incubate for 5-10 min to allow rehydration of the lipids. Vortex the lipid solution for about 30 s to form MLVs.
  12. Make 0.5-1 mL aliquots of the MLVs in 1.5 mL microcentrifuge tubes. Plunge the tubes in liquid nitrogen, seal with a transparent film, and store at −20 °C (for up to 6 weeks).
    **NOTE:** Lipid stock concentrations are chosen to allow sufficiently large volumes that allow reliable pipetting using the Hamilton syringes. If the volumes necessary to make the stock are too large to dissolve the dried lipid powder, make multiple dilutions of the stock to ensure reproducible mixing of various lipids.
10. Preparation of small unilamellar vesicles (SUVs)
  1. Take out an aliquot of MLVs from the −20°C storage and thaw it at room temperature. Flash freeze the vesicles by plunging the microcentrifuge tube in liquid nitrogen for 15-30 s and immediately put it in a water bath set at 45 °C until the solution has thawed completely (1-2 min). Repeat the above freeze-thaw cycle 10x-15x until the solution looks less turbid.
    **NOTE:** Set the temperature of the water bath higher than the transition temperature of the lipid mix being thawed to allow uniform lipid mixing.
  2. Equilibrate a syringe-based mini extruder fitted with an 80 nm pore size polycarbonate filter membrane with SUV rehydration buffer. Ensure that there is no leakage or bubbles in the system. While the extrusion method yields monodisperse SUVs with minimum lipid damage, lipid mixes with negative charge can stick to the polycarbonate membrane.
  3. Gently pass the thawed lipid solution through the pre-equilibrated extruder from one side to the other and then back. Repeat the cycle 5x-10x until the lipid solution turns visibly clear, indicating the formation of SUVs with ~100 nm diameter.
  4. Centrifuge the extruded suspension (or tip-sonicate the solution; see note below) at 15,000 x *g* for 60 min at 4 °C to pellet down the lipid debris. Collect the top 80% of the solution without disturbing the pellet and without creating bubbles. Transfer the supernatant containing the SUVs to a fresh microcentrifuge tube and store on ice for up to 6 days.
    **NOTE:** An alternative to centrifugation is tip sonication performed as follows. Turn on a microtip sonicator and set the following settings: Amplitude = 30% of the maximum, ON time = 10 s, OFF time = 60 s. Clean the tip of the micro-sonicator with deionized water followed by 2 N NaOH, chloroform, and again deionized water. Dip the sonicator tip in each of these solutions and sonicate for 1-2 cycles using the above settings. Dip the clean tip in the freeze-thawed vesicle solution and sonicate for 3-6 cycles on ice until the solution turns clear.
  5. After centrifugation, check for signs of high lipid degradation or a failed lipid extrusion as the formation of a thin whitish film and/or a clearly visible pellet. In these cases, do not proceed and repeat the SUV preparation steps again.
    **NOTE:** The shelf life of SUVs can differ for different lipid mixtures. SUVs made of DOPC: DGS-NTA-Ni^2+^are stable for up to 6 days for the purposes of these experiments. Tips to resolve common problems can be found in Table 2.

### 2 Reconstitution of membrane-tethered actin networks

**Figure 3:**
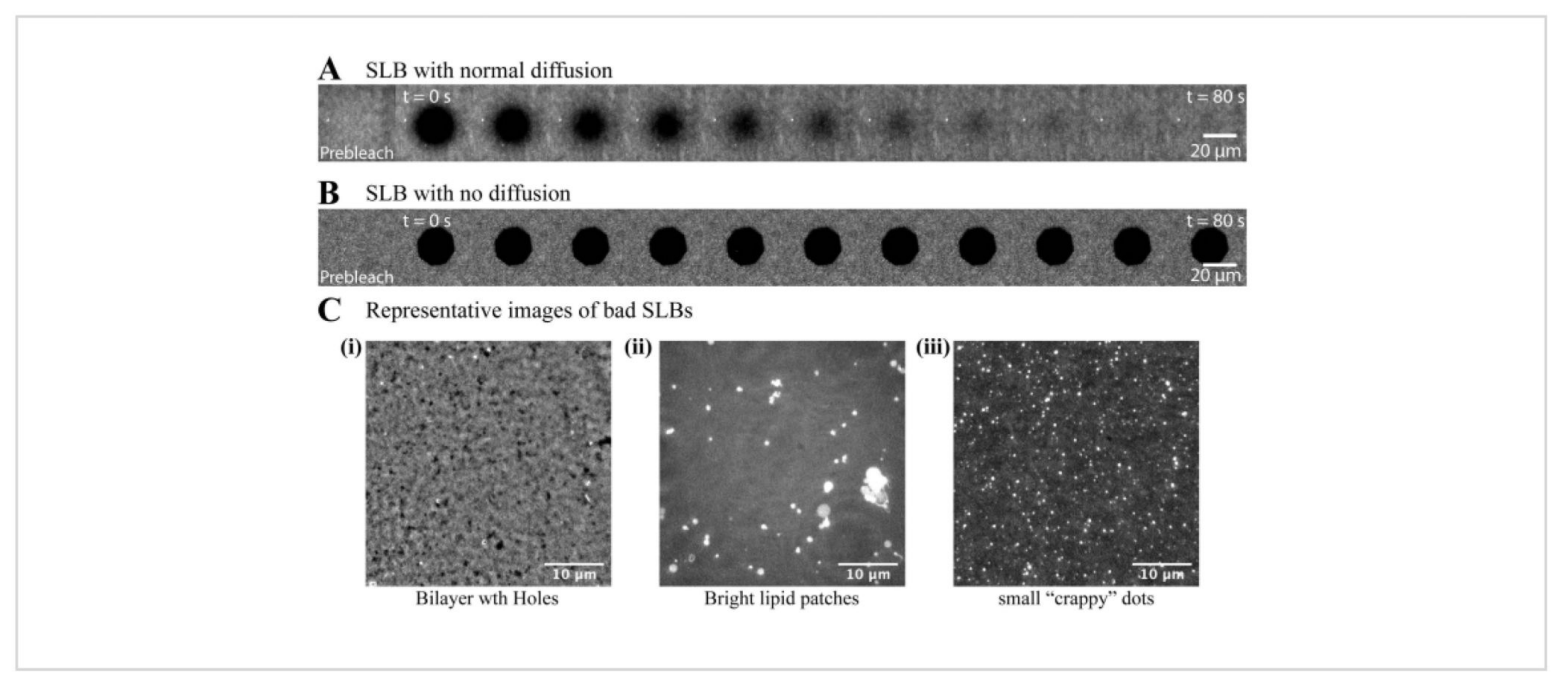
Quality assessment of the bilayers with quick FRAP assay. Supported lipid bilayers (SLBs) prepared from DOPC and Ni-NTA lipids (98:2 mol%) are coated with HYE (10xHis-YFP-tagged membrane-actin linker). After the unbound protein is washed out, the fluorescent bilayer is imaged under a TIRF microscope. A small region on the bilayer is photobleached with high laser power, and the recovery of fluorescence is recorded. (**A**) A good bilayer always recovers fast, with an expected diffusion coefficient of 1-1.5 μm^2^/s for the lipid composition used in this case. (**B**) Bad bilayers recover very slowly or do not recover at all. (**C**) Representative images of bad bilayers: (**C-i**) a bilayer with holes, (**C-ii**) a bilayer with big, immobile lipid patches, and (**C-iii**) a bilayer with small, immobile dots. Please click here to view a larger version of this figure.

**Figure 4:**
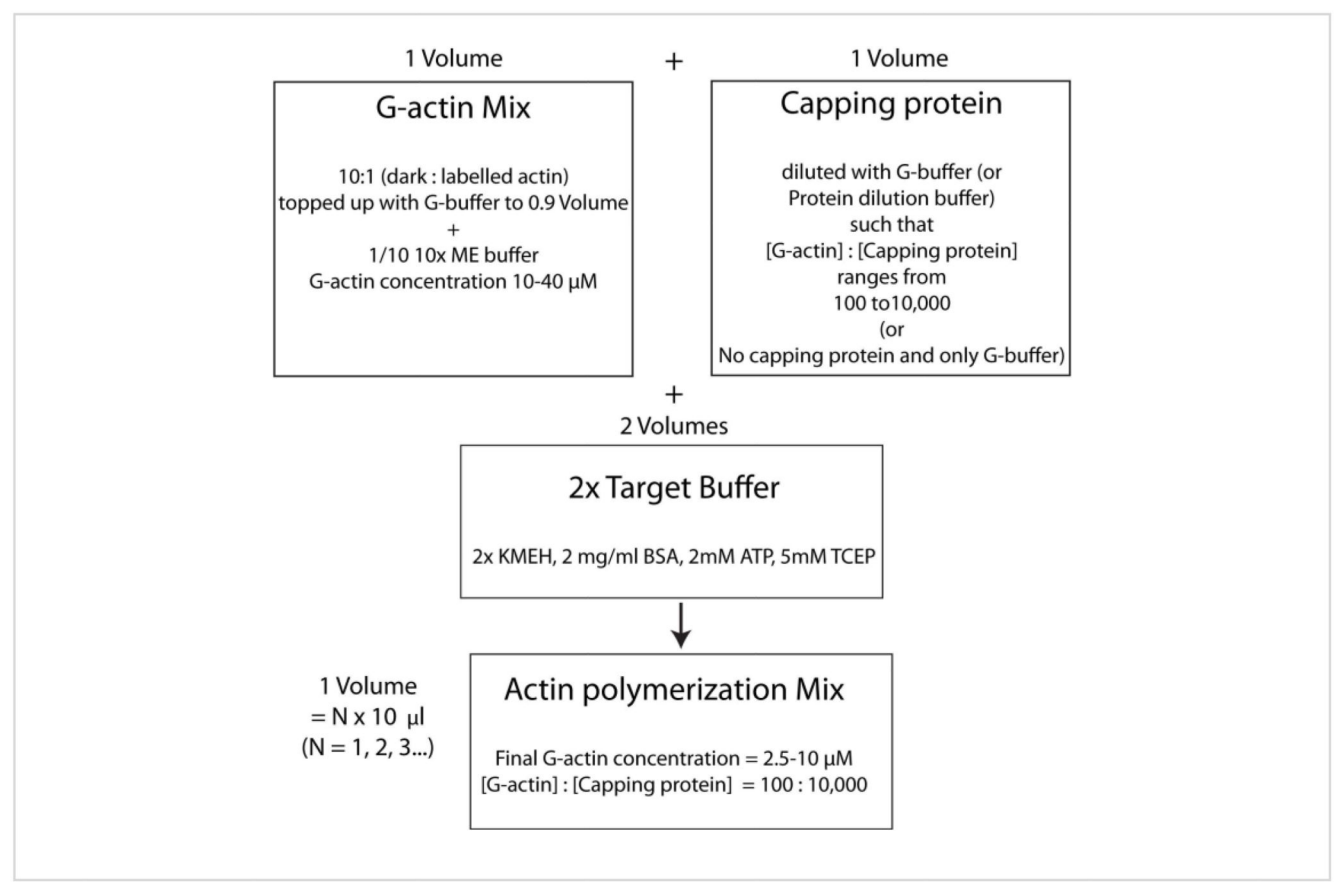
Schematic showing how to polymerize actin using the target buffer method. Please click here to view a larger version of this figure.

**Figure 5:**
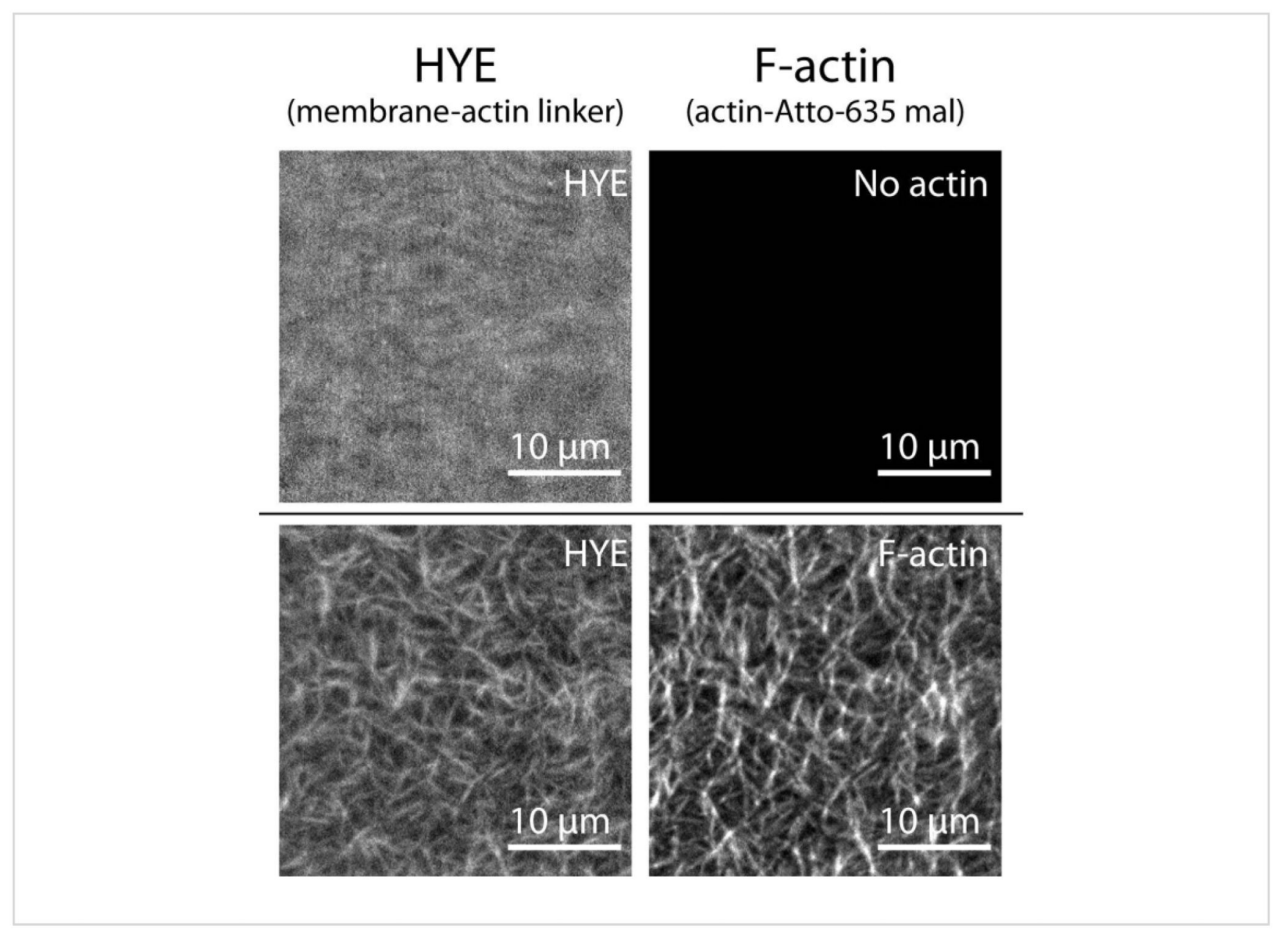
Spatial organization of HYE upon binding to F-actin. TIRF snapshots showing the spatial organization of HYE before and after the addition of actin filaments (labeled with Atto-635 maleimide). The HYE organization is homogenous before the addition of F-actin and becomes colocalized and coaligned along actin filaments. Please click here to view a larger version of this figure.

1. Preparation of sample chambers
  1. Take 3-5 rectangular glass coverslips and place them inside a Coplin jar. Turn on the bath sonicator and set the temperature to 65 °C. Fill the Coplin jar with 2% cleaning solution to fully submerge the coverslips and place it in the sonicator for 30 min at full pulse mode.
  2. Use blunt PTFE-coated forceps to remove the coverslips one by one from the jar. Rinse them thoroughly with distilled water and place them in another Coplin jar filled with 2 N NaOH.
  3. Sonicate the coverslips for 20 min at full pulse mode. Remove the coverslips one by one, rinse thoroughly with distilled water, and place in another Coplin jar filled with distilled water.
    **NOTE:** Optionally, sonicate the coverslips in distilled water for 20 min and then rinse them again with distilled water.
  4. Immediately before starting the experiment, take the jar containing the coverslips in a chemical hood fitted with an N_2_gas supply.
  5. Optimize the air pressure of the N_2_gas stream by trial and error so that it is just enough to displace water from the coverslip surface without breaking it. Align the flow of N_2_gas parallel to the coverslip plane to reduce the possibility of breaking the coverslip.
  6. Use gloves and forceps to remove the coverslips one by one from the jar to dry them under the N_2_stream. Dry both the sides of each coverslip and place them on a clean plastic grid with a cover. Place the box with the coverslips in a desiccator to avoid contact with dust particles in the air.
    **NOTE:** The N_2_-dried coverslips can be stored in a desiccator where they can remain hydrophilic for up to 2 days. This strategy might be useful when many bilayers are required for the experiment or if the experiment takes longer than 8 h.
  7. Take autoclaved PCR tubes and cut out their lids and lower conical halves with a sharp surgical blade. Take the cylindrical half-cut tubes one by one, apply UV curable adhesive to the smooth rim of each cut tube, and place it inverted on a freshly cleaned coverslip such that the rim sits flat on the coverslip.
  8. Do not move the cylinder laterally once it is positioned on the coverslip to ensure the glue does not spill to the central space of the chamber. Rectangular coverslips can comfortably accommodate up to three reaction chambers, and the round ones can accommodate only one in the center (Figure 1).
  9. Put the chamber-bearing coverslips inside a UV ozone cleaner with an O_2_supply and vacuum (or use a UV illuminator). Turn on the UV light and illuminate for 3-5 min to allow the adhesive to polymerize. Perform longer illumination (10-15 min) to improve the hydrophilicity of the cover glass and, hence, the quality of the lipid bilayer.
  10. Store the dry UV-illuminated sample chambers for up to 8 h inside small plastic boxes (such as empty rectangular coverslip boxes) wrapped in transparent film to reduce contact with dust particles in the air.
    **NOTE:** A steady stream of O_2_in the presence of UV light forms ozone and oxygen radicals that can remove organic impurities from the surface of the coverslip. A vacuum will prevent the leakage of toxic ozone that is formed during the process.
  11. Take out the coverslips and test the chambers for leakage by filling them up with distilled water. Each chamber can hold up to ~150 μL of sample. Discard the leaky chambers.
    **NOTE:** Another great and safe cleaning option is the plasma cleaner. The time and power settings depend on the model, but make sure not to overtreat the glass slides with plasma as this will result in a reduction of lipid mobility. The surface treatment can affect the mobility of lipids^27^, as has been observed with prolonged treatment with the cleaning solution (>45 min) or NaOH (>30 min).
2. Preparation of supported lipid bilayers
  1. Wash each chamber with SLB formation buffer (or 1x PBS) to remove any surface contaminants, leaving 100 μL of buffer at the end. Mark the level of the buffer at 100 μL with a permanent marker to reproducibly track changes in the volume.
  2. Add 2 μL of 0.1 M CaCl_2_to the chamber. This improves the adsorption of the vesicles to the glass surface, enhancing the bilayer formation in the next step. Add 8 μL of the SUV solution (from step 1.10.) to each chamber and incubate for 15 min at 25 °C.
    **NOTE:** The volume of the SUV mix to be added can be estimated by calculating the total number of lipids (with an average area of 0.72 nm^2^) that are needed to completely cover the exposed hydrophilic area of the well with two lipid layers.
  3. Wash off the unbound vesicles with actin motility buffer (1x KMEH). First, remove 50 μL of the SLB formation buffer, leaving only 50 μL in the sample chamber. Second, add 100 μL of 1x KMEH to the chamber. Mix gently and then remove 100 μL of the buffer without touching the bottom.
    **NOTE:** It is important to be gentle while washing. Make sure the pipette tip does not touch the bottom of the chamber. Keep the pipette inclined to direct the flow of buffer to the wall of the chamber and not directly at the bilayer, as a direct flow may disrupt the bilayer. Be careful to not introduce any air bubbles while pipetting as air may reach the lipid bilayer and cause defects in it.
  4. Repeat the washes 10x by adding 100 μL of 1x KMEH and removing 100 μL.
  5. Add 10 μL of 1 mg/mL β-Casein to the bilayer, mix gently and incubate for 5-10 min. β-casein blocks the regions on the coverslip where the bilayer has not formed. Wash off β-casein 3x with 1x KMEH as described in step 2.2.3. and bring the buffer level back to the 100 μL mark.
3. Addition of membrane-actin linker
  1. During the β-casein incubation (step 2.2.5.), take out an aliquot of membrane-actin linker protein from −80 °C, thaw it quickly at 37 °C, and then keep it on ice. Dilute the aliquot with protein dilution buffer to a concentration of 1 μM.
  2. Add the linker protein at a defined final concentration (typically 5-20 nM) and mix gently. To ensure a rapid equilibration of the protein in the chamber, add volumes that are larger than 20 μL by premixing the linker protein with 1x KMEH.
  3. Incubate for 40 min at room temperature. Wash 3x with 1x KMEH buffer to remove the unbound HSE protein (as in step 2.2.3.). Bring the buffer level in each chamber back to the 100 μL mark. The sample is now ready for imaging.
4. Quality assessment of the lipid bilayer
  **NOTE:** This is an optional step that does not have to be performed every time. We recommend this assessment be performed every time fresh SUVs are made from frozen MLV stocks.
  1. Turn on the microscope, the excitation lasers, and the detection cameras. Make sure the laser is aligned, the objective is cleaned, and the software is ready to acquire images.
  2. Put oil on the 100x objective, mount the sample on the microscope stage, and focus the objective on the bilayer. Make sure the laser position is such that it undergoes total internal reflection on the sample. Use a 488 nm excitation laser to check the fluorescence intensity distribution of the bilayer-bound 10xHis-YFP-EzrinABD.
    **NOTE:** Good quality bilayers show a large-scale, uniform distribution of fluorescence intensity. Bad bilayers show intense and patchy fluorescent spots.
  3. To determine the integrity of the bilayer, perform a FRAP assay.
    1. Select a region of interest on the bilayer and record a few images of the field of view using imaging conditions that provide a signal-to-noise ratio of 5:1 or higher. Pause the recording and close the field diaphragm of the TIRF microscope to focus a concentrated laser beam on a small circular region of the bilayer to locally bleach the fluorophores.
    2. Turn on the laser to its maximum output to photobleach the small region for 3-10 s and then turn the laser off. Reopen the field diaphragm to its original radius, readjust the imaging condition back to (pre-bleach) settings, and immediately resume to record the recovery of fluorescent signal in the field of view.
  4. Check if the bilayer is fluid. Good bilayers with normal lateral diffusion recover fast, while bad bilayers recover slowly or do not recover at all (Figure 3). If the bilayer is not recovering, check the troubleshooting section and restart. Save the images as 16-bit TIFF files. For a quantitative estimation of the diffusion coefficient, check step 3. below.
  5. Polymerization of fluorescent actin
    **NOTE:** To save time, start polymerizing actin during the incubation time of the HSE protein binding to the bilayer (step 2.3.) or during the quality assessment of the bilayer (step 2.4.).
    1. Mix unlabeled and fluorescently labeled G-actin in a 10:1 molar ratio and top it up with G-Buffer so that the concentration of G-actin is 20 μM. The concentration at which actin is finally polymerized will be 1/4 of this value. Add 1/10 of 10x ME buffer to the mix for a 1x solution and incubate for 2 min. This step replaces the Ca^2+^ions bound to G-actin with Mg^2+^ions. Ensure the final volume is in multiples of 10 μL.
    2. Add the desired amount of capping protein as follows. Thaw a vial of capping protein stock quickly at 37 °C and then keep it on ice. Dilute with G-buffer such that the concentration of capping protein now is twice its desired final concentration in the polymerization mix. Add an equal volume of the diluted capping protein solution to the actin mix from step 2.5.1.
    3. Finally, add an equal volume of fresh 2x target buffer to the reaction mix. The final volume of the solution should be four times the volume of the actin mix at the end of step 2.5.2. Ensure the final concentration of KMEH is 1x, of ATP is 1 mM, of BSA is 1 mg/mL, and of G-actin is 5 μM.
      Incubate in the dark at 25 °C for 45-60 min to allow polymerization to happen.
      **NOTE:** This is called the target buffer strategy, in which one volume of Mg^2+^G-actin (step 2.5.1.) is mixed with one volume of capping protein mix (step 2.5.2.) and two volumes of 2x target buffer (step 2.5.3.). This makes it easier to scale up or down the amount of actin and to alter the relative concentration of capping protein (or any other actin modulator; Figure 4).
  6. Addition of fluorescent actin filaments
    1. Cut a few 200 μL tips with a sharp blade or scissors to make them blunt ended. Gently pipette out the required volume of 5 μM polymerized actin (from step 2.5.3.) with a blunt-ended pipette tip (to prevent shearing of actin filaments) and add it to a clean autoclaved PCR tube.
    2. Add 1x KMEH to the tube to make the volume >20 μL and mix gently to avoid shearing of F-actin. From the mounted sample chamber, remove an equal volume of the buffer.
    3. Add the polymerized actin solution to the chamber and gently pipette up and down 3x without touching the bilayer at the bottom. This allows actin filaments to uniformly distribute on the bilayer. Mount the sample on the TIRF microscope (see step 2.4.1. and step 2.4.2.).
    4. One can record the process of F-actin binding to the bilayer. Incubate for 20-30 min. Record a few images from different fields of view after F-actin addition has reached a steady state. Observe change in the spatial organization of 10xHis-YFP-EzrinABD before (homogeneous) and after actin organization.
      **NOTE:** HYE is uniformly distributed over the lipid bilayer in the absence of actin. Upon addition of actin filaments, HYE colocalizes with F-actin. The extent of colocalization depends on the actin-binding affinity of the linker protein; the stronger the affinity, the higher the colocalization and the slower the lateral mobility of the linker protein (Figure 5).
  7. Addition of myosin II
    1. After 30 min of actin incubation, mount the sample back on the microscope (if it was unmounted). Check the signal in the linker protein and F-actin channels. Adjust the imaging conditions if needed.
    2. Select a good region with uniform linker protein signal and uniformly scattered actin filaments and no artifacts for a long time-lapse recording. Record 10-15 frames at 0.1-0.2 Hz before myosin addition and pause the recording. Pipette out the required volume of recycled muscle myosin-II from the stock vial with a blunt-ended pipette tip (to prevent the shearing of myosin filaments) and add to a clean autoclaved PCR tube.
    3. Immediately add 1x KMEH to the tube to make the volume >20 μL and mix gently. One can also add ATP, ATP regenerating mix, photo-stabilizing agents, etc. during this step. Carefully remove an equal volume of the buffer from the mounted sample chamber without disturbing it.
    4. Gently add the myosin solution to the sample chamber. Do not pipette up and down as it will disturb the surface-bound filaments. Immediately resume the time-lapse recording and observe the system as it evolves from the pre-myosin state to ATP-fueled contractile acto-myosin flows and aster formation to an ATP-depleted jammed state (see representative results).
    5. Take background images for all the channels using a buffer only sample. Save all the images as 16-bit.tiff files. See Table 2for tips to resolve common problems.

### 3 Data analysis

1. Using Fiji software (https://imagej.net), subtract the background from the linker protein images (from step 2.4.). Measure the mean intensity values from the bleached spot and a reference region.
2. Normalize the time traces from the bleached spot and the reference region to the intensity of their respective pre-bleach intensity values. Divide each timepoint in the normalized bleached region values by the respective time points in the normalized reference region time trace. Correct the resultant normalized time trace for background and for any systematic fluctuations in intensity during acquisition (global photobleaching, z-drift, etc.).
  1. Use a fitting-free, manual method^28^to estimate the diffusion coefficient of the bilayer tethered proteins. Briefly, the half time of the recovery profile, *τ*_1/2_, can be calculated by looking at the time when the normalized recovery profile reaches half of its steady state:
    (1) $$F({\text{τ}}_{\frac{1}{2}})=F_{\frac{1}{2}}=\frac{F_{0}+F_{\infty }}{2}$$
    Here, *F*_0_is the mean intensity in the bleached region in the first frame after photobleaching, and *F*_∞_is the long-term steady state value of recovery of the bilayer.
  2. Estimate the effective bleach radius, *r*_*e*_, a parameter that corrects for diffusion during photobleaching, from a line scan of the post bleach spot profile^29^. The half width at half minimum of this line-scan passing through the center of the bleach spot, *r*_1/2_, relates to *r*_*e*_as follows:
    (2) $$r_{e}=r_{1/2}\sqrt{\frac{2}{\text{ln}2}}≃1.7r_{1/2}$$
    The *τ*_1/2_calculated in step 2.8.3., *r*_*e*_calculated in step 2.8.4., and the originally set bleach radius, *r*_*n*_, are used to calculate the diffusion coefficient (*D*) using the following formula:
    (3) $$D=\frac{r_{e}^{2}+r_{n}^{2}}{8\tau_{\frac{1}{2}}}$$
3. Image analysis of actomyosin asters
  1. Using Fiji, subtract the background from all the recorded images in all the channels. Correct the images for any non-uniform illumination or interference pattern using flat-field correction.
    **NOTE:** One can use colored plastic slides, which are good flat samples to do such corrections. For the linker protein and actin filaments on a planar bilayer, one can also use the average projection of multiple pre-myosin images to create channel-specific illumination correction maps.
    1. For the HYE channel, shown here, take an average intensity projection of multiple HYE images (recorded from different regions of the lipid bilayer before myosin addition). Apply an appropriate Gaussian filter (*σ*= 50 pixels to 80 pixels) to the average projection (from pre-myosin images or any standard flat sample).
    2. Convert the filtered image to a 32-bit image. Divide all the pixel values by the mean of the whole image. This will give a normalized correction map for the HYE channel. Divide all the images in the HYE channel with this map for flat-field correction. Create correction maps for other channels using the same strategy.
  2. Correct for photobleaching using an exponential or simple ratio method (depending on the intensity decay profile) in Fiji.
    1. To correct for any temporal x-y misalignment (translational movement), merge all the photobleach-corrected channels into a single Hyperstack. Using the Hyperstack-Reg plugin in Fiji, apply a Rigid Body or Translation transformation.
    2. Finally, split the aligned Hyperstack into individual channels and save them separately as 16-bit TIFF stacks for further analysis.

## Representative Results

**Figure 6:**
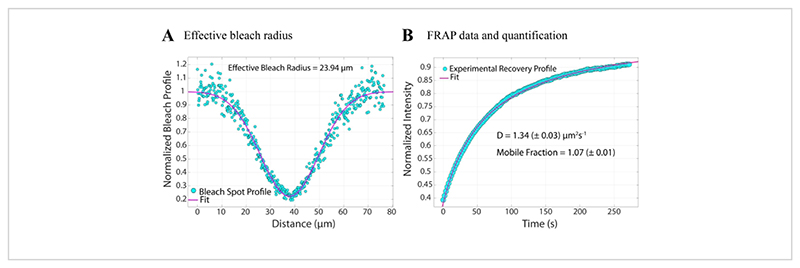
Quantifying the diffusion coefficient of lipid bilayers. (**A**) Line profile of the first image after photobleaching (t = 0 s in Figure 3A) and its fit to equation 4to calculate the effective bleach radius. (**B**) The recovery profile of the bleached region and its fit to equation 5to calculate the diffusion coefficient and mobile fraction. Please click here to view a larger version of this figure.

For representation, here a typical postbleach profile from the 1st image after photobleaching (image at t = 0 s in Figure 3A) and its fit to the following function^28^(see Figure 6A) is shown:

(4) $$f(r)=1-K\text{exp}(-\frac{2r^{2}}{r_{e}^{2}})$$

The value of *r*_*e*_(23.94 μm) calculated by the fit to this curve is very similar to the *r*_*e*_calculated in step 2.8.4. (23.24 μm). Here, *K* is a bleach depth parameter that can be directly estimated from *F*_0_(described in step 2.8.4.). Similarly, Figure 6Bshows the recovery profile and its fit to the following function^28^:

(5) $$F(t)=\{1-\frac{K}{1+\frac{r_{n}^{2}}{r_{e}^{2}}+\frac{8Dt}{r_{e}^{2}}}\}*M_{F}+(1-M_{F})F_{0},$$

We find the fitted value of the diffusion coefficient to be 1.34 μm^2^/s, a value that closely agrees with the value of 1.39 μm^2^/s that is calculated by the formula in step 2.8.4. Here, *M*_*F*_stands for the mobile fraction of the lipid bilayer that represents the fraction of the bleached population that recovers back. The mobility of lipid-anchored molecules depends, of course, on the lipid composition and its physical state (liquid or gel phase). For our experiments using DOPC-based lipid membranes, the mobility should be >1 μm^2^/ s, and the mobile fraction should not be less than 0.9 to indicate a good lipid bilayer. We recommend the use of the manual fitting-free method for a quick test of the quality and mobility of the bilayer. The fitting method can be useful while automating the analysis for many FRAP curves. Further, if one wants to perform a more sophisticated FRAP experiment to systematically characterize diffusion in the system, we recommend the reader to this review from Lorén et al.^30^for more detail on fitting models and potential pitfalls in experimental design.

**Figure 7:**
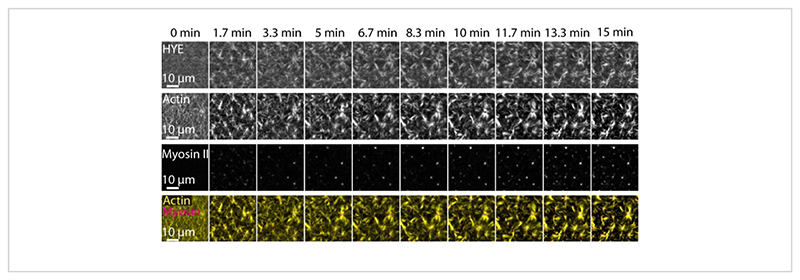
Contractile actomyosin flows drive local clustering of the membrane-actin linker protein HYE. TIRF snapshots of HYE (YFP-tagged), actin filaments (labeled with Atto-635 maleimide), and myosin II filaments (labeled with Atto-565 maleimide) upon addition of myosin II to an SLB containing HYE and F-actin. Time is indicated on the top: 0 min is immediately before fluorescent myofilaments started appearing in the TIRF field. HYE and F-actin are homogeneously distributed over the lipid bilayer prior to myosin addition (0 min). Myosin activity induces contractile actomyosin flows, which emerge into aster-like structures at the steady state (15 min), driving local clustering of the coupled membrane component (HYE). The lowest row is a merge of actin (yellow) and myosin II (magenta) images showing the organization of actin and myosin at different time points. The images used in making these montages were corrected in Fiji for background signal, non-uniform intensity patterns, and translational movement. Scale bar = 10 μm. For details, see Supplementary Video S1. Please click here to view a larger version of this figure.

A typical result of the experiments described above showing the dynamic assembly and organization of an acto-myosin network linked on a supported lipid bilayer imaged by TIRF microscopy is depicted in Figure 7and Supplementary Video S1.

Figure 7shows an image montage of the linker protein, F-actin, and myosin-II.

## Discussion

This protocol presents a versatile platform and a starting point to design experiments to study the membrane-cortex interface of cells. Critical steps are the preparation of clean glass slides, using fresh lipids for efficient SUV formation (both affecting the quality of SLBs), and the use of freshly recycled myosin II proteins for dynamic actin filament reorganization. When imaging dynamics over a long time, it is very important to incorporate an oxygen scavenger system (e.g., protocatechuic acid and protocatechuate 3 4-dioxygenase^5, 31^).

The open-chamber design allows the sequential addition of components to an existing system without inducing lipid flows. This can be an important advantage over commonly used, closed-chamber approaches or work using encapsulated proteins within liposomes^36^. Contrary effects such as protein-induced membrane deformation cannot be studied with glass-adsorbed lipid bilayers.

The lipid bilayers can be formed with a wide range of lipid compositions. It begins with adsorption of the lipid vesicles to the hydrophilic glass surface, followed by either spontaneous vesicle rupture due to surface-vesicle and direct vesicle-vesicle interactions or the adsorbed vesicles reaching a critical coverage after which a small fraction of vesicles rupture, forming active edges, which eventually leads to the bilayer formation^32^. Besides glass, various substrates can be used to form supported lipid bilayers, such as Mica (e.g., for atomic force microscopy), soft substrates (e.g., poly-di-methyl-siloxane), polymer cushions^33, 34, 35^, spanning between holes of electron microscopy grids^14^. Droplet interface bilayers are another interesting method to create stable, free-standing lipid bilayers^36^. The inclusion of acto-myosin networks into vesicles or emulsions is a very powerful method to study this minimal system in a cell-like geometry^37, 38^, and which is described in detail elsewhere^39^.

## Supplementary Material

Supplementary Video

## Figures and Tables

**Table 1:** List of buffer compositions used in this protocol.

Buffer Name	Composition
Lipid Rehydration buffer	50 mM HEPES, 150 mM NaCl, 5% sucrose, pH 7.5
SLB Formation buffer	50 mM HEPES, 150 mM NaCl, pH 5-6
SLB Storage buffer	50 mM HEPES, 150 mM NaCl, pH 7.2
Protein Dilution buffer	20 mM HEPES, 100 mM KCl, 1mM TCEP or DTT, pH 7.2
1X ME or Actin ion-exchange buffer	50 mM MgCl2, 0.2 mM EGTA, 10 mM HEPES, pH 7.2 (store at 4°C)
1X KMEH or Actin polymerization buffer	50 mM KCl, 1 mM MgCl2, 1 mM EGTA, 50 mM HEPES, pH 7.2
100 mM ATP stock	100 mM ATP disodium salt, 50 mM Tris, 50 mM NaCl, 5 mM MgCl2, 2 mM EGTA, pH 7.5 (store at -20°C)
2x Target buffer	2x KMEH, 2 mg/ml BSA, 2mM ATP, 5mM TCEP (stored at 4°C)
G-buffer	2 mM Tris, 0.1 mM CaCl2, 0.2 mM ATP, 0.5 mM TCEP, 0.04 % NaN3, pH 8 (store at 4°C)
Myosin II buffer	500 mM KCl, 1 mM EDTA, 10-20 mM Hepes, pH 7.0
Gel-filtration chromatography buffer	50 mM Tris-HCl, 150-300 mM NaCl, 5 mM TCEP, 0.1% Tween-20, pH 7.5
Capping protein Storage buffer	10 mM Tris·Cl, 50 mM NaCl, 1 mM TCEP, pH 7.5, 20% glycerol

**Table 2:** Troubleshooting guide summarizing common problems and corresponding solutions.

Common problems and their trouble shooting	Problem	Cause	Possible solutions
**1**	**Lipid bilayer shows no diffusion**	The most probable cause for this problem is dirty coverglass that can happen when the cleaning solution is aged or the heating did not take place during bath sonication. These bilayers have a ‘vesicular’ appearance because the bursted vesicles stick to the coverglass but do not fuse with each other. Using MLVs older than 6 weeks or SUVs older than 6 days, or adding low amounts of SUVs can also lead to vesicular bilayer formation.	Use fresh cleaning solution. Make sure the heater is turned on and the temperature is between 45-65°C. Use fresh lipid mixes. (Using a fluorescent lipid probe versus a fluorescent protein probe can sometimes manifest differently. E.g., if the bilayer has sub-diffraction defects and surface passivation step is skipped (or does not work), the lipid probe will show a uniform intensity distribution but the fluorescent protein probe may display bright fluorescent spots.)
**2**	**Lipid bilayer has bright patches**	Long incubation of SUVs for bilayer formation can create a lipid bilayer that is overall diffusing but with occasional bright patches. These patches can be multi-layered bilayers that can attract large amounts of fluorescent probe.	15-20 min incubation with SUVs is enough. Make sure the probe is not aggregating: a quick hard spin of the linker protein (300 x g for 15 min at 4 °C) can remove the aggregates
**3**	**Lipid bilayer has dark holes**	This happens when the bilayer is made from old SUVs and imaged for prolonged hours (> 4 hours post formation), or the pH of the solution changes drastically due to prolonged imaging (e.g., in the high ATP state and in the presence of certain oxygen scavengers), or when the surface is over-passivated with beta-Casein (adding too much beta-Casein for more than 10-15 min and or not washing it out).	Use fresh lipids. Reduce the imaging frame rate or the effective laser illumination time. Use buffers with higher buffering capacity.
**4**	**Lipid bilayer shows slow diffusion**	Lipid bilayers with high percentage of cholesterol, long saturated lipids or charged lipids diffuse slower.	In such cases, prepare your sample at a high temperature. One can also use a simple, tested lipid composition as a control along with complex and untested lipid compositions. Make sure the glass is clean.
**5**	**Actin does not polymerize**	Target buffer is old, G-actin stock is too old, old and new G-actin were co-polymerized.	Make sure the Ca^2+^ is replaced by Mg^2+^ before polymerization (using ME buffer). Use fresh ATP-Mg^2+^ stock. Use freshly recycled G-actin. Make sure the concentration of F-actin (in terms of G-actin) added to the bilayer is higher than 0.2 μM. For lower concentrations, use phalloidin stabilized F-actin.
**6**	**Actin does not bind to the bilayer**	Membrane-actin linker is not added or added at very low concentration—this can be inferred form the fluorescence of the linker protein. If the fluorescence is decent, the membrane-actin linker has lost actin-binding capacity. Also, if the linker protein is non-specifically bound to the glass surface (when the bilayer is bad), it might not recruit actin filaments.	Make sure the bilayer is diffusing. Use fresh linker protein
**7**	**Fluorescent F-actin signal is weak**	The ratio of labelled to dark actin is too low. Either the labelled actin or unlabelled actin is too old and they are not copolymerizing with each other.	Recycle actin again, and retry the ploymerization with freshly recycled actin. Photodamage can destroy or depolymerize F-actin; if possible, use red or far-red dyes for actin (and myosin).
**8**	**Myosin does not show contractility**	it may be observed that after adding ATP to the myosin-infused system, there is no contractility of the acto-myosin.	Check if myosin concentration or purity level is good. Use freshly recycled myosin (use within 6 weeks after recycling). Adding fresh ATP to the myosin mix can help. De-gassing buffers and using oxygen scavengers etc. can reduce photodamaging of the motors. Further information might be found in the protocols by Plastino et al. or Stam et al. of the same methods collection
**9**	**Coverglass is not hydrophilic**	Coverglass is not cleaned properly.	Clean hydrophilllic coverglass is crucial for the lipid bilayer formation. A useful, visual readout of the hydrophilicity of the coverglass after the cleaning protocol is to observe the wetting of the glass by water. Add a small volume of water to a flat-lying coverslip. The water will remain in the shape of a round droplet if the coverslip is not cleaned properly. However, the same volume of water will spread out and form a thin layer, on a treated hydrophilic coverglass. This wetting behaviour of the water on the cover glass surface can be used to ascertain if the cleaning steps with the cleaning solution/NaOH have worked.
