# Supplementary material for: Reconstitution of Membrane-tethered Minimal Actin Cortices on Supported Lipid Bilayers
Source: J Vis Exp. Author manuscript; Available in PMC 2024 Sep 20. (PMC7616522; doi:10.3791/63968)
Supplement: Supplementary Video [file EMS198599-Supplementary-Supplementary_Video.pdf]

Supplementary Video S1: Contractile actomyosin flows drive local clustering of the membrane-actin linker protein HYE. TIRF timelapse of HYE (YFP-tagged), actin filaments (labeled with Atto-635 maleimide), and myosin II filaments (labeled with Atto-565 maleimide) upon addition of myosin II to an SLB containing HYE and F-actin. Time is indicated on the top: 0 min is immediately before fluorescent myofilaments started appearing in the TIRF field. Scale bar = 10 µm. Please click here to download this File.

Copyright © 2022 JoVE Creative Commons Attribution 3.0 License
